# Information-Theoretic Dual Adaptive Control Revisited: Multivariable Extension with Applications to Fault-Tolerant Control

**DOI:** 10.3390/e28030304

**Published:** 2026-03-09

**Authors:** Joseph-Julien Yamé

**Affiliations:** Centre de Recherche en Automatique de Nancy (CRAN), National Centre for Scientific Research (CNRS), UMR 7039, Campus Sciences, Université de Lorraine, 54506 Vandoeuvre-lès-Nancy, France; joseph.yame@univ-lorraine.fr

**Keywords:** dual adaptive control, fault-tolerant control, information theory, multivariable systems, convex optimization, actuator faults

## Abstract

This paper revisits and extends the information-theoretic dual adaptive control framework initially developed by the author for single-input single-output systems to multiple-input multiple-output (MIMO) systems, with specific application to fault-tolerant control (FTC). The core contribution is a MIMO formulation that preserves the essential dual property, i.e., balancing control performance against parameter learning, while addressing the increased complexity of coupled multivariable systems. A convexity condition is derived for the MIMO optimization problem, generalizing the original SISO condition. The framework naturally handles actuator faults through a parameter vector that includes effectiveness factors, with fault detection achieved via monitoring of information gain. Control reconfiguration strategies ensure graceful performance degradation under faults. Simulation results demonstrate the effectiveness of this dual approach to FTC methods in balancing detection speed, identification accuracy, and tracking performance, while maintaining computational feasibility for real-time implementation.

## 1. Introduction

Adaptive control has long addressed one of the fundamental challenges in control theory, i.e., managing systems with unknown or time-varying parameters [1,2,3,4,5]. This class of problems inherently involves a fundamental trade-off between achieving regulation and improving parameter estimates, a dilemma first formalized by Feldbaum in his seminal work on dual control [6]. Classical adaptive schemes update controller parameters based on online measurements, yet they often struggle to balance exploitation, aimed at maintaining desired performance, with exploration, which seeks to enhance system knowledge and reduce uncertainty.

An elegant solution to this problem was proposed by the author in [7,8], who introduced an information-theoretic formulation of dual adaptive control for SISO stochastic systems. By incorporating an information measure directly into the cost function, the control law explicitly accounted for both tracking performance and parameter estimation quality. This approach led to a cubic equation describing the optimal control and provided theoretical guarantees of convergence and uniqueness. However, while conceptually powerful, this original formulation was restricted to single-input single-output (SISO) systems and thus could not address the complexities of modern multivariable (MIMO) systems. In such systems, the coupling between inputs and outputs significantly complicates both estimation and control, and these challenges are magnified in safety-critical domains requiring fault tolerance. Indeed, with the growing emphasis on resilience in aircraft, autonomous vehicles, and industrial processes, fault-tolerant control (FTC) has become a crucial design requirement, reinforcing the need to extend dual adaptive control to multivariable settings.

The evolution of dual control since Feldbaum’s early work has been rich and diverse. Early stochastic dynamic programming approaches [9,10,11] established solid theoretical foundations but soon proved computationally impractical for real-time use. To address this limitation, various suboptimal formulations were proposed [12,13,14,15], trading optimality for feasibility through simplifying assumptions. The author’s information-theoretic framework [7] marked a conceptual shift by embedding information gain within the control objective, providing a systematic way to manage uncertainty. For MIMO systems, however, adaptive control poses additional difficulties, including parameter coupling, higher dimensionality, and more stringent stability conditions [3,5]. Parallel advances in FTC [16,17] have traditionally relied on modular architectures that separate fault detection, isolation, and reconfiguration, often resulting in delays or uncoordinated responses. Recent progress in convex optimization [18] and computational capability makes it possible to revisit dual control ideas and develop tractable formulations that integrate fault tolerance directly within the control scheme [19]. Moreover, recent research has explored FTC in even more complex dynamical structures, such as fault-tolerant synchronization of switched complex networks [20], optimized fault-tolerant control for switched complex networks [21], and fuzzy tracking control for Markov jump systems confronting mismatched faults [22], highlighting the diverse challenges in modern control applications.

Building on these insights, this paper extends the dual control framework [7] from SISO to MIMO systems by introducing a vectorized representation of model parameters and matrix-valued information measures. The core contributions of this paper are threefold:

(i) **Theoretical extension:** We provide a complete MIMO formulation that preserves the essential dual property—balancing control performance against parameter learning—while addressing the increased complexity of coupled multivariable systems through vectorized representations and matrix-valued information measures.
(ii) **Generalized convexity condition:** A novel convexity condition (Theorem 2) is derived for the MIMO optimization problem, generalizing the original SISO condition and guaranteeing a unique, globally optimal dual control solution, thereby making the problem tractable for online computation.
(iii) **Integrated FTC framework:** Fault detection, identification, and reconfiguration are unified within a single online optimization problem, eliminating the need for separate diagnostic layers and enabling faster, more coordinated responses to actuator faults. The information gain serves simultaneously as a learning objective and a fault indicator.

The convex nature of the optimization problem enables online computation of the MIMO control using efficient solvers. Comprehensive MATLAB simulations for a toy MIMO system demonstrate the real-time feasibility of the proposed approach and its advantages over conventional FTC architectures.

The paper is organized as follows. Section 2 revisits the original SISO dual controller and summarizes its theoretical foundations. Section 3 develops the MIMO extension and presents the associated convexity analysis. Section 4 formulates the integrated FTC methodology, while Section 5 provides numerical experiments and implementation details. Finally, Section 6 concludes the paper and outlines future research directions.

## 2. Review of the Information-Theoretic Dual Adaptive SISO Controller

This section reviews the key contributions of the original work of the author [7], which introduced an information-theoretic framework for dual adaptive control of single-input single-output (SISO) stochastic systems.

### 2.1. Problem Formulation

Consider the SISO discrete-time stochastic system described by the following autoregressive with exogenous input (ARX) model:

(1) $$y\left(k\right)+a_{1}\left(k\right)y\left(k-1\right)+\ldots +a_{n}\left(k\right)y\left(k-n\right)=b_{1}\left(k\right)u\left(k-1\right)+\ldots +b_{n}\left(k\right)u\left(k-n\right)+e\left(k\right)$$

where y(k)∈R is the system output, u(k)∈R is the control input, and e(k) is a Gaussian white noise disturbance with zero mean and variance $\sigma^{2}$, independent of past inputs and outputs. The system parameters $\theta \left(k\right)={\left[b_{1}\left(k\right),b_{2}\left(k\right),\ldots ,b_{n}\left(k\right),a_{1}\left(k\right),\ldots ,a_{n}\left(k\right)\right]}^{⊤}$ are time-varying and stochastic, evolving according to the random walk model

(2) θ(k+1)=θ(k)+w(k)

with w(k) being zero-mean Gaussian white noise with covariance matrix R. The initial parameter vector θ(0) is Gaussian with mean $\theta_{0}$ and covariance $P_{0}$. Define the regressor vector

(3) $$h^{⊤}\left(k\right)=\left[u\left(k-1\right),u\left(k-2\right),\ldots ,u\left(k-n\right),-y\left(k-1\right),\ldots ,-y\left(k-n\right)\right]$$

then, (1) reads

(4) $$y\left(k\right)=h^{⊤}\left(k\right)\theta \left(k\right)+e\left(k\right).$$

The key innovation in [7] is the introduction of an information-theoretic measure to quantify parameter uncertainty and explicitly embed learning into the control objective. Let $Y^{k}=\left\{y\left(0\right),y\left(1\right),\ldots ,y\left(k\right),u\left(0\right),\ldots ,u\left(k-1\right)\right\}$ denote the information available at time *k*. The conditional entropy [23] of the system parameters given $Y^{k}$ is defined as

(5) $$H\left(\theta |Y^{k}\right)=-E\left[\log p(\theta |Y^{k})\right]$$

where $p(\theta |Y^{k})$ is the conditional probability density function of θ given $Y^{k}$. The information gained about the system parameters from the observations up to time *k* is then measured by the reduction in entropy with respect to the initial uncertainty,

(6) $$I\left(\theta ,Y^{k}\right)=H\left(\theta |Y^{k}\right)-H\left(\theta \left(0\right)\right).$$

For the Gaussian system (1) and (2), the conditional distribution $p(\theta \left(k\right)|Y^{k})$ is Gaussian with mean $\hat{\theta }\left(k\right)$ and covariance P(k), both updated recursively by a Kalman filter. In this case, the information measure simplifies to

(7) $$I\left(\theta ,Y^{k}\right)=\frac{1}{2}\left[\log\;\det P_{0}-\log\;\det P\left(k\right)\right].$$

Within this framework, the dual control objective is to balance control performance and parameter learning in a single optimization problem. This trade-off is formalized through the one-step cost function

(8) $$J\left(u\left(k\right)\right)=E\left[{\left(y\left(k+1\right)-y_{r}\left(k+1\right)\right)}^{2}\;|\;Y^{k}\right]-\gamma I\left(\theta ,Y^{k+1}\right)$$

where $y_{r}\left(k+1\right)$ is the reference signal, γ>0 is a learning coefficient that weights the importance of parameter learning relative to control performance, and $Y^{k+1}=Y^{k}\cup \left\{y\left(k+1\right),u\left(k\right)\right\}$. Using the properties of the Kalman filter and the Gaussian information measure (7), the cost function can be rewritten, up to an additive constant independent of u(k), as

(9) $$J\left(u\left(k\right)\right)=E\left[{\left(y\left(k+1\right)-y_{r}\left(k+1\right)\right)}^{2}\;|\;Y^{k}\right]-\frac{\gamma }{2}\log\left[\frac{1}{1+h^{⊤}\left(k+1\right)P\left(k\right)h\left(k+1\right)}\right]+\mathrm{constant}.$$

The resulting optimization problem thus captures in a unified manner the competing goals of accurate tracking and informative excitation.

### 2.2. Optimal Solution

The optimal control $u^{*}\left(k\right)$ minimizes the cost function (9). Taking the derivative with respect to u(k) and setting it to zero yields the following key result:

**Theorem** **1** (Existence and Uniqueness [7])**.** *Assuming the condition*

(10) $$0<\gamma <1+h^{⊤}\left(k+1\right)P\left(k\right)h\left(k+1\right)$$

*holds at each time k, the cost function J(u(k)) has a unique minimum. This minimum satisfies the cubic equation:*

(11) $$A\left(k\right)u^{3}\left(k\right)+B\left(k\right)u^{2}\left(k\right)+C\left(k\right)u\left(k\right)+D\left(k\right)=0$$

*where the coefficients A(k), B(k), C(k), and D(k) are time-varying functions of the parameter estimates and covariance matrix (see Appendix A of [7] for explicit expressions).*

The convexity condition (10) guarantees that this cubic equation has exactly one real root, which corresponds to the globally optimal dual control input. In practice, this cubic equation can be solved efficiently and robustly at each time step using a simple numerical root-finding algorithm, such as Newton’s method, which converges quickly given the convexity property and a good initial guess (e.g., the previous control input u(k−1)). An insightful closed-form expression for $u^{*}\left(k\right)$ can be obtained by introducing the normalized learning coefficient

(12) $$\gamma_{k}=\frac{\gamma }{1+h^{⊤}\left(k+1\right)P\left(k\right)h\left(k+1\right)}.$$

With this definition, the optimal control law can be written as

(13) $$u^{*}\left(k\right)=\frac{{\hat{b}}_{1}\left(k+1\right)y_{r}\left(k+1\right)-z^{⊤}\left(k+1\right)\left[{\hat{b}}_{1}\left(k+1\right)\hat{g}\left(k+1\right)+P_{21}\left(k\right)\left(1-\gamma_{k}\right)\right]}{{\hat{b}}_{1}^{2}\left(k+1\right)+P_{11}\left(k\right)\left(1-\gamma_{k}\right)}$$

where ${\hat{b}}_{1}\left(k+1\right)$ is the estimate of the first input coefficient, $\hat{g}\left(k+1\right)={\left[{\hat{b}}_{2}\left(k+1\right),\ldots ,{\hat{b}}_{n}\left(k+1\right),{\hat{a}}_{1}\left(k+1\right),\ldots ,{\hat{a}}_{n}\left(k+1\right)\right]}^{⊤}$ collects the remaining estimated parameters, $z^{⊤}\left(k+1\right)=\left[u\left(k-1\right),\ldots ,u\left(k-n+1\right),-y\left(k\right),\ldots ,-y\left(k-n+1\right)\right]$ is a modified regressor vector, and $P_{11}\left(k\right)$ and $P_{21}\left(k\right)$ are appropriate partitions of the covariance matrix P(k). This expression makes explicit how the learning coefficient influences the control action through the covariance structure and the regressor. The structure of (13) also clarifies the interpretation of the dual control law. When $\gamma_{k}=0$, there is no explicit emphasis on learning, and the control law reduces to the cautious controller [13], which accounts for parameter uncertainty but does not actively probe the system. In contrast, when $\gamma_{k}=1$, learning is given maximum weight and the controller becomes equivalent to the certainty-equivalence controller or self-tuning regulator [1], which ignores parameter uncertainty and relies solely on current estimates. For intermediate values $\gamma_{k}\in \left[0,1\right]$, the dual controller continuously interpolates between these two extremes, naturally balancing caution (control performance) and curiosity (parameter learning) within a single analytic expression. Beyond its instantaneous behavior, the dual controller also exhibits appealing asymptotic properties. Under standard assumptions of consistent parameter estimation, namely $\hat{\theta }\left(k\right)\to \theta$ and P(k)→0 as k→∞, the dual control law (13) converges to the minimum-variance control law:

(14) $$\lim_{k\to \infty }u^{*}\left(k\right)=\frac{{\hat{b}}_{1}y_{r}\left(k+1\right)-z^{⊤}\left(k+1\right)\hat{g}}{{\hat{b}}_{1}^{2}}.$$

This result confirms that, once the parameters are accurately identified and the covariance matrix has collapsed, the dual effect vanishes and the controller focuses exclusively on optimal regulation.

From an implementation standpoint, the dual adaptive controller can be realized with modest computational effort, making it suitable for real-time applications as demonstrated in [7]. At each sampling instant, the algorithm updates the parameter estimates $\hat{\theta }\left(k\right)$ and covariance P(k) using a Kalman filter, computes the optimal control input either by solving the cubic Equation (11) or by evaluating the closed-form expression (13), and then applies this control to the system before updating the regressor vector. The learning coefficient γ can be chosen offline or adapted online to ensure satisfaction of the convexity condition (10), thereby preserving uniqueness and stability of the solution while tuning the balance between performance and excitation. For later references, the dual adaptive control algorithm and a summary of the key results are given in Algorithm 1 and Table 1.
**Algorithm 1** Dual Adaptive Control Algorithm [7]  1:**Initialize:** $\hat{\theta }\left(0\right)$, P(0), γ  2:**for** k=0,1,2,…**do**  3:      **Step 1:** Measure y(k)  4:      **Step 2:** Update Kalman filter:  5:       $\;\hat{\theta }\left(k+1\right)=\hat{\theta }\left(k\right)+K\left(k\right)\left(y\left(k\right)-h^{⊤}\left(k\right)\hat{\theta }\left(k\right)\right)$  6:       $\;P\left(k+1\right)=P\left(k\right)-K\left(k\right)h^{⊤}\left(k\right)P\left(k\right)$  7:      **Step 3:** Compute $u^{*}\left(k\right)$ using (13)  8:      **Step 4:** Apply $u^{*}\left(k\right)$ to system  9:      **Step 5:** Update regressor vector h(k+1)10:**end for**

This section has reviewed the fundamental contributions of the original work of the author, setting the stage for the multivariable extension developed in the following sections.

## 3. MIMO System Formulation and Cost Function Extension

### 3.1. MIMO System Modeling and Fault Representation

Consider a *p*-input, *m*-output discrete-time stochastic system described by the MIMO ARX model:

(15) $$y\left(k\right)+\sum_{i=1}^{n_{a}}A_{i}\left(k\right)y\left(k-i\right)=\sum_{i=1}^{n_{b}}B_{i}\left(k\right)\Lambda \left(k-i\right)u\left(k-i\right)+e\left(k\right)$$

where $y\left(k\right)\in R^{m}$ is the output vector, $u\left(k\right)\in R^{p}$ is the input vector, $A_{i}\left(k\right)\in R^{m\times m}$ and $B_{i}\left(k\right)\in R^{m\times p}$ are coefficient matrices, and $e\left(k\right)∼N\left(0,\Sigma_{e}\right)$ is Gaussian white noise. Actuator faults are modeled through the effectiveness matrix $\Lambda \left(k\right)=\mathrm{diag}(\lambda_{1}\left(k\right),\ldots ,\lambda_{p}\left(k\right))$, where $\lambda_{j}\left(k\right)\in \left[0,1\right]$ represents the effectiveness of the *j*-th actuator. The operator $\mathrm{diag}(a_{1},\ldots ,a_{n})$ refers to the square diagonal matrix having $a_{i}$ as its *i*-th diagonal entry, for i=1,…,n.

Define the regression vector

(16) $$h_{s}\left(k\right)={\left[\begin{array}{cccccc} -y^{⊤}\left(k-1\right) & \ldots & -y^{⊤}\left(k-n_{a}\right) & u^{⊤}\left(k-1\right) & \ldots & u^{⊤}\left(k-n_{b}\right) \end{array}\right]}^{⊤}.$$

and consider the fault-free MIMO system, i.e., when the actuator effectiveness matrix $\Lambda \left(k\right)=I_{p}$. Introduce the nominal system parameter vector $\theta_{s}\in R^{n_{s}}$ ($n_{s}=m^{2}n_{a}+mpn_{b}$) as the vectorization of matrices $A_{i}$ and $B_{i}$:

(17) $$\theta_{s}={\left[\begin{array}{cccccc} \mathrm{vec}{\left(A_{1}\right)}^{⊤} & \ldots & \mathrm{vec}{\left(A_{n_{a}}\right)}^{⊤} & \mathrm{vec}{\left(B_{1}\right)}^{⊤} & \ldots & \mathrm{vec}{\left(B_{n_{b}}\right)}^{⊤} \end{array}\right]}^{⊤}.$$

Using the identity $\mathrm{vec}\left(AX\right)=\left(X^{⊤}⊗I\right)\mathrm{vec}\left(A\right)$, where ⊗ is the Kronecker product, the fault-free MIMO ARX model can be written in regression form as

(18) $$y\left(k\right)=H_{s}^{⊤}\left(k\right)\theta_{s}+e\left(k\right)$$

where the regressor matrix $H_{s}\left(k\right)\in R^{n_{s}\times m}$ is given by

(19) $$H_{s}^{⊤}\left(k\right)=h_{s}^{⊤}\left(k\right)⊗I_{m}.$$

Now suppose that the MIMO system experiences failures in the actuators. The model (15) can then be rewritten as

(20) $$y\left(k\right)-\sum_{i=1}^{n_{a}}A_{i}\left(k\right)y\left(k-i\right)-\sum_{i=1}^{n_{b}}B_{i}\left(k\right)u\left(k-i\right)=\sum_{i=1}^{n_{b}}B_{i}\left(k\right)\left(\Lambda -I_{p}\right)u\left(k-i\right)+e\left(k\right)$$

where it is assumed that the actuator efficiency matrix Λ(k−j) is time-independent and takes on a constant value Λ different from the identity matrix. Equation (20) reads concisely

(21) $$z\left(k\right)=\sum_{i=1}^{n_{b}}B_{i}\left(k\right)\Delta u\left(k-i\right)+e\left(k\right)$$

where z(k) represents the left-hand side of Equation (20) and

$$\Delta =\Lambda -I_{p}=\mathrm{diag}\left(\delta_{1},\ldots ,\delta_{p}\right)$$

with $\delta_{i}=\lambda_{i}-1$, for i=1,…,p. Let $\delta ={\left[\delta_{1},\delta_{2},\ldots ,\delta_{p}\right]}^{T}$, and since Δ is diagonal, we have

$$B_{i}\left(k\right)\;\Delta \;u\left(k-i\right)=B_{i}\left(k\right)\left(\delta ⊙u(k-i)\right).$$

where ⊙ denotes the Hadamard (element-wise) product. The above operation consists of scaling each column of $B_{i}\left(k\right)$ by the corresponding element of u(k−i), which is equivalent to $B_{i}\left(\delta ⊙u(k-i)\right)=B_{i}\;\mathrm{diag}\left(u(k-i)\right)\;\delta .$ Then, Equation (21) can written as a regression

(22) $$z\left(k\right)=H_{\delta }^{T}\left(k\right)\;\delta +e\left(k\right),$$

where the fault regressor matrix that has exactly *p* columns is given by $H_{\delta }^{T}\left(k\right)=\sum_{i=1}^{n_{b}}B_{i}\left(k\right)\;\mathrm{diag}\left(u(k-i)\right)$ and can be written explicitly as

(23) $$H_{\delta }^{T}\left(k\right)=\left[\begin{array}{cccc} h_{\delta ,1}\left(k\right) & h_{\delta ,2}\left(k\right) & \ldots & h_{\delta ,p}\left(k\right) \end{array}\right]$$

The *l*-th column (l=1,…,p) of this fault regressor matrix equals to

$$h_{\delta ,l}\left(k\right)=\sum_{i=1}^{n_{b}}u_{l}\left(k-i\right)\;b_{\left(:,l\right)}^{i}\left(k\right)\in R^{m},$$

with vector $b_{\left(:,l\right)}^{i}\left(k\right)$ denoting the *l*-th column of the matrix $B_{i}\left(k\right)$. Note that this fault regressor matrix depends on the nominal parameter $\theta_{s}$ through its dependence on the elements of matrices $B_{i}$.

Finally, the MIMO ARX model (15) can be rewritten as

(24) $$y\left(k\right)=H_{s}^{⊤}\left(k\right)\theta_{s}+H_{\delta }^{⊤}\left(k\right)\delta +e\left(k\right)$$

**Remark** **1.** *The formulation (24) exhibits a bilinear structure: while δ appears linearly, the regressor $H_{\delta }$ depends on the system nominal parameters $\theta_{s}$. This coupling makes clearly the joint parameters $\left(\theta_{s},\delta \right)$ not structurally identifiable [24], as it can also be seen from the product terms $B_{i}\Lambda$ in the model (15).*

With regards to Remark 1, the identification of the nominal system parameter $\theta_{s}$ will be decoupled from the estimation of the actuators’ effectiveness parameter δ. This approach is a practical one that initially determines the nominal behavior of the system (in the absence of faults) so that fault occurence can be detected and fault magnitude estimation can be performed to achieve fault-tolerant control. From this two-stage estimation, the healthy operation of the system will be concerned with estimation of $\left(A_{i},B_{i}\right)$ when $\Lambda =I_{p}\;\left(i.e.,\;\delta =0\right)$ and, for actuator faulty behaviors, only δ will be estimated with fixed ’nominal’ estimated system parameter ${\hat{\theta }}_{s}$.

### 3.2. Parameter Estimation and Information Measure for MIMO Systems

The modeling of the parametric uncertainties of the MIMO system will be described here using a stochastic evolution model, specifically a random walk given by

(25) θ(k+1)=θ(k)+w(k)

with w(k)∼N(0,Q), and initial Gaussian distribution $\theta \left(0\right)∼N\left(\theta_{0},P_{0}\right)$, Q and $P_{0}$ being the covariance matrices of w and $\theta_{0}$, respectively. In Equation (25), parameter θ represents either $\theta_{s}$ under nominal conditions or the actuator effectiveness δ under faulty conditions. A Kalman filter is employed for parameter estimation. Based on the available information at time *k*, $Y^{k}=\left\{y\left(0\right),\ldots ,y\left(k\right),u\left(0\right),\ldots ,u\left(k-1\right)\right\}$, the parameter vector θ(k) is conditionally Gaussian-distributed with mean $\hat{\theta }\left(k\right)$ and covariance P(k), both updated through the following recursion:

(26) $$\begin{array}{cc} \hat{\theta }\left(k|k-1\right) & =\hat{\theta }(k-1|k-1)\; \end{array}$$

(27) $$\begin{array}{cc} P(k|k-1) & =P(k-1|k-1)+Q\; \end{array}$$

(28) $$\begin{array}{cc} \;K(k) & =P\left(k|k-1\right)H\left(k\right){\left[H^{⊤}\left(k\right)P\left(k|k-1\right)H\left(k\right)+\Sigma_{e}\right]}^{-1} \end{array}$$

(29) $$\begin{array}{cc} \;\hat{\theta }\left(k|k\right) & =\hat{\theta }\left(k|k-1\right)+K\left(k\right)\left[y\left(k\right)-H^{⊤}\left(k\right)\hat{\theta }\left(k|k-1\right)\right] \end{array}$$

(30) $$\begin{array}{cc} P(k|k) & =\left[I-K\left(k\right)H^{⊤}\left(k\right)\right]P\left(k|k-1\right) \end{array}$$

where H(k) is either (19) or (23) depending on the context.

The information-theoretic measure of the SISO case, (6) and (7), extends naturally to MIMO through the conditional entropy of the parameter vector. For the Gaussian system, the information gained simplifies to

(31) $$I\left(\theta ,Y^{k}\right)=\frac{1}{2}\left[\log\det P_{0}-\log\det P\left(k\right)\right].$$

This measure possesses key properties: coordinate invariance, additivity for independent parameters, non-negativity, and monotonic non-decrease with additional observations under standard conditions. It serves simultaneously as a learning objective, fault detection signal, and convergence metric.

### 3.3. MIMO Dual Control Cost Function and Optimization

The dual control objective for MIMO systems seeks, as in the SISO case, to balance control performance against parameter learning, and this is formalized through the cost function,

(32) $$J_{\mathrm{MIMO}}\left(u\left(k\right)\right)=E\left[∥y_{r}\left(k+1\right)-y\left(k+1\right){∥}_{Q}^{2}|Y^{k}\right]+u^{⊤}Ru-\gamma I\left(\theta ,Y^{k+1}\right)$$

where $y_{r}\left(k+1\right)$ is the reference signal, ${∥\cdot ∥}_{Q}$ denotes a weighted norm with Q≻0, R≻0 is the control weight matrix, and γ>0 is the learning coefficient. Using Kalman filter properties, this simplifies to

(33) $$J_{\mathrm{MIMO}}\left(u\right)=J_{p}\left(u\right)-\frac{\gamma }{2}J_{i}\left(u\right)$$

where

(34) $$\begin{array}{cc} J_{p}\left(u\right) & =E[∥y_{r}{-y∥}_{Q}^{2}\left|Y^{k}\right]+u^{⊤}Ru\\ J_{i}\left(u\right) & =\log\det\left(I+H^{⊤}PH\Sigma_{e}^{-1}\right) \end{array}$$

Detailed computation of the conditional expectation term in $J_{p}$ gives

(35) $$E\left[∥y_{r}\left(k+1\right)-y\left(k+1\right){∥}_{Q}^{2}|Y^{k}\right]={∥y_{r}-\hat{y}∥}_{Q}^{2}+\mathrm{tr}\left(QP_{y}\right)$$

where $\hat{y}=H^{⊤}\hat{\theta }$ and $P_{y}=H^{⊤}PH+\Sigma_{e}$. It is worth noting that the term $\mathrm{tr}\left(QP_{y}\right)$ represents the uncertainty penalty. It increases with larger parameter uncertainty (larger P), as well as with larger measurement noise (larger $\Sigma_{e}$), and through $P_{y}=H^{⊤}PH+\Sigma_{e}$, it depends on both the parameter covariance P and the regressor H (which depends on u). This term inherently encourages *exploration*, i.e., controls that reduce P (by making $H^{⊤}PH$ smaller) will reduce this cost. The information gain term $-\frac{\gamma }{2}\log\det\left(I+H^{⊤}PH\Sigma_{e}^{-1}\right)$ provides additional exploration incentive, explicitly rewarding controls that maximize information about the parameters.

Now, the optimal dual control problem can be formulated as follows:

(36) $$\begin{array}{cccc} \; & \underset{u}{\mathrm{minimize}} & \; & J_{\mathrm{MIMO}}\left(u\right)\\ \; & \mathrm{subject}\;\mathrm{to} & \; & u_{\min}⪯u⪯u_{\max}\\ \; & \; & & {∥u-u\left(k-1\right)∥}_{\infty }\leq \Delta_{\max}\\ \; & \; & & Cu⪯d \end{array}$$

where the last constraint represents additional linear constraints on the control inputs. The following proposition provides a key step toward a tractable solution to (36).

**Proposition** **1** (Convexity Preservation Under Constraints)**.**
*If the constraint set $U=\{u\in R^{p}:u_{\min}⪯u⪯u_{\max},∥u\left(k\right)-u\left(k-1\right){∥}_{\infty }\leq \Delta_{\max},Cu⪯d\}$ is compact and convex, and $J_{\mathrm{MIMO}}\left(u\right)$ is convex on U, then the optimization problem (36) is convex.*

Recall that in the SISO case (see Section 2 and the original paper [7]), a condition on the learning factor γ was instrumental in ensuring the convexity of the objective function and thus the uniqueness of the dual control solution through the resolution of a cubic equation.

We are now in a position to state the main result of this paper.

**Theorem** **2** (Convexity Condition of the Dual Control Cost)**.**
*Let the dual control cost function $J_{\mathrm{MIMO}}\left(u\right)$ be given by (32) with $J_{p}\left(u\right)$, with $J_{i}\left(u\right)$ defined by (33) and (34). Define the Hessian matrices:*

(37) $$G\left(u\right)=\nabla_{u}^{2}J_{p}\left(u\right),\;L\left(u\right)=\nabla_{u}^{2}J_{i}\left(u\right).$$

*Both are symmetric matrices as Hessians of real-valued functions.*

*Let U be the convex set of admissible controls, and assume*

(38) $$c=\underset{u\in U}{\inf}\lambda_{\min}\left(G\left(u\right)\right)>0,\;M=\underset{u\in U}{\sup}{∥L\left(u\right)∥}_{2}<\infty .$$

*Then, the cost function $J_{\mathrm{MIMO}}\left(u\right)$ is strictly convex on U if*

(39) $$0<\gamma <\frac{2c}{M}.$$

**Proof.**  See Appendix A.    □

**Corollary** **1.** *Suppose that the assumptions of Theorem 2 hold. Assume in addition that there exists a constant $\underline{\lambda }>0$ such that*

(40) $$\lambda_{\min}\;\left(H{\left(u\right)}^{⊤}PH\left(u\right)\right)\geq \underline{\lambda },\;\forall u\in U.$$

*Then, there exists a constant C>0, independent of u, such that a sufficient condition for strict convexity of the cost function $J_{MIMO}\left(u\right)$ on U is*

(41) $$0<\gamma \leq C\left(1+\lambda_{\min}{(H\left(u\right)}^{⊤}PH\left(u\right)\right).$$

**Proof.**  See Appendix A.    □

**Remark** **2.** *The above corollary shows that the admissible exploration or probing weight γ scales linearly with the minimum eigenvalue of the information matrix $\lambda_{\min}{(H\left(u\right)}^{⊤}PH\left(u\right)$. Condition (41) generalizes the SISO result (10), i.e., the larger $\lambda_{\min}{(H\left(u\right)}^{⊤}PH\left(u\right)$, the richer the information and the more strictly convex the cost becomes. Hence, the upper bound on γ can be larger. Note, however, that the bound is conservative in the multivariable case due to the use of uniform spectral norm bounds on the Hessian of the log-determinant term.*

**Remark** **3** (Challenges in the MIMO extension). *Extending the original SISO dual control framework to MIMO systems entails several non-trivial challenges. First, the coupling between inputs and outputs makes the regressor matrix H(k) a function of all past inputs and outputs, which significantly complicates the structure of the information measure and the resulting cost function. Second, the dimensionality of the parameter vector grows rapidly (from 2n in SISO to $m^{2}n_{a}+mpn_{b}$ in MIMO), which not only increases the computational burden but also raises identifiability issues, especially when actuator faults are present. Third, while the information gain $I(\theta ,Y^{k})$ remains a scalar quantity (as it is defined via the logarithm of a determinant), its expression involves the determinant of a matrix that depends on the regressor H. This makes the analysis of convexity considerably more intricate than in the SISO case, where a simple scalar term $h^{⊤}Ph$ appears (compare Theorem 2 and Corollary 1 with the original condition (10)). Finally, the bilinear coupling between the nominal parameters and the fault vector δ (see Remark 1) creates a structural identifiability problem that forces us to adopt a two-stage estimation strategy. Thus, while the underlying information-theoretic philosophy is preserved, the MIMO formulation is far from a straightforward extension of the SISO case and requires dedicated theoretical developments, as presented in this paper.*

The convexity conditions developed above provide the foundation for a computationally efficient implementation. In the next subsection, the complete MIMO dual adaptive controller is summarized in algorithmic form integrating parameter estimation, convexity-preserving adaptation of γ, and constrained optimization into a single online procedure.

### 3.4. Implementation and Properties of the MIMO Dual Controller

A complete implementation of the MIMO dual adaptive controller is provided in Algorithm 2. The algorithm maintains the essential dual property, balancing control performance against parameter learning, while addressing the complexities of multivariable systems. Key properties of the algorithm include the following: (i) convexity enforcement via γ-adaptation (Step 4, Theorem 2), ensuring a unique global minimum; (ii) computational tractability through convex optimization solvers (interior-point or SQP [25]) with warm-starting; (iii) generalization of SISO properties, reducing to Algorithm 1 for scalar systems; (iv) preservation of the information-theoretic foundation via the matrix log-determinant term, which extends the SISO information measure.

**Parameter Selection:** Condition (39) is a theoretical bound; in practice, we enforce a local convexity margin $c_{\mathrm{target}}$ as described. The convexity margin $c_{\mathrm{target}}$ and adaptation factor η are tuning parameters with clear practical interpretations. The parameter $c_{\mathrm{target}}$ ensures the Hessian of $J_{MIMO}\left(u\right)$ remains positive definite with minimum eigenvalue $\geq c_{\mathrm{target}}$, guaranteeing strict convexity and numerical stability. For initial implementation, we recommend $c_{\mathrm{target}}=10^{-4}\cdot \max\left(\mathrm{diag}\left(R\right)\right)$ and η=0.7. These provide a conservative convexity guarantee while allowing sufficient exploration. In practice, $c_{\mathrm{target}}$ should be small relative to the typical minimum eigenvalue of $\nabla_{u}^{2}J_{p}\left(u\right)$ (which is bounded below by $2\lambda_{\min}\left(R\right)$), while η controls how aggressively γ is reduced when convexity is threatened. These parameters can be fine-tuned via simulation to balance exploration rate against numerical robustness. The condition in Step 4 checks whether the current γ maintains the desired convexity margin $c_{\mathrm{target}}$. If violated, γ is reduced by factor η to restore convexity. This local verification and adjustment provides an online, practical implementation of the theoretical condition (39). If the condition is satisfied, γ remains unchanged, allowing persistent exploration when the convexity margin is satisfied.

Table 2 summarizes the main points of the MIMO dual control and shows that its formulation inherits the asymptotic properties of the SISO controller: as parameter uncertainty vanishes (P(k)→0), the dual effect diminishes and the controller converges to a certainty-equivalent multivariable regulator. This convergence, coupled with the online convexity guarantee, ensures both stability and performance.

**Table 2:** Summary of MIMO Dual Control Framework.

Concept	MIMO Formulation
System Model	$y\left(k\right)=H^{⊤}\left(k\right)\theta \left(k\right)+e\left(k\right)$
Parameter Evolution	θ(k+1)=θ(k)+w(k),w(k)∼N(0,Q)
Information Measure	$I\left(\theta ,Y^{k}\right)=\frac{1}{2}\left[\log\det P_{0}-\log\det P\left(k\right)\right]$
Dual Cost Function	$J_{\mathrm{MIMO}}\left(u\right)=E[∥y_{r}{-y∥}_{Q}^{2}\left|Y^{k}\right]+u^{⊤}Ru-\gamma I\left(\theta ,Y^{k+1}\right)$
Convexity Condition	$0<\gamma <\frac{2c}{M}$ (Theorem 2), with $c=\inf\lambda_{\min}\left(G\left(u\right)\right)$, $M={\sup∥L\left(u\right)∥}_{2}$
Optimal Control	Solution of $\min_{u\in U}J_{\mathrm{MIMO}}\left(u\right)$ (constrained convex optimization)
Asymptotic Behavior	Converges to LQR-type controller as P(k)→0

**Algorithm 2** MIMO Dual Adaptive Control**Require:** MIMO system model (15), performance weights Q≻0, R≻0, initial learning coefficient $\gamma_{0}>0$**Ensure:** Dual control sequence $\left\{u^{*}\left(k\right)\right\}$  1:**Offline initialization:**  2:Set constraints $u_{\min}$, $u_{\max}$, $\Delta_{\max}$, C, d  3:Initialize Kalman filter: $\hat{\theta }\left(0|0\right)=\theta_{0}$, $P\left(0|0\right)=P_{0}$  4:Choose convexity margin $c_{\mathrm{target}}>0$, adaptation factor η∈(0,1)  5:$\gamma \leftarrow \gamma_{0}$  6:**for** k=0,1,2,… **do**  7:       **Step 1:** Measure output y(k)  8:       **Step 2:** Update Kalman filter:  9:       $\hat{\theta }\left(k|k-1\right)\leftarrow \hat{\theta }\left(k-1|k-1\right)$10:       P(k|k−1)←P(k−1|k−1)+Q11:       $K\left(k\right)\leftarrow P\left(k|k-1\right)H\left(k\right){\left[H^{⊤}\left(k\right)P\left(k|k-1\right)H\left(k\right)+\Sigma_{e}\right]}^{-1}$12:       $\hat{\theta }\left(k|k\right)\leftarrow \hat{\theta }\left(k|k-1\right)+K\left(k\right)\left[y\left(k\right)-H^{⊤}\left(k\right)\hat{\theta }\left(k|k-1\right)\right]$13:       $P\left(k|k\right)\leftarrow \left[I-K\left(k\right)H^{⊤}\left(k\right)\right]P\left(k|k-1\right)$14:       **Step 3:** Form dual cost function for u=u(k):15:       $\hat{y}\leftarrow H^{⊤}\left(k+1\right)\hat{\theta }\left(k|k\right)$16:       $P_{y}\leftarrow H^{⊤}\left(k+1\right)P\left(k|k\right)H\left(k+1\right)+\Sigma_{e}$17:       $J_{\mathrm{MIMO}}\left(u\right)\leftarrow {∥y_{r}-\hat{y}∥}_{Q}^{2}+\mathrm{tr}\left(QP_{y}\right)+u^{⊤}Ru-\frac{\gamma }{2}\log\det\left(I+H^{⊤}\left(k+1\right)P\left(k|k\right)H\left(k+1\right)\Sigma_{e}^{-1}\right)$18:       **Step 4:** Enforce convexity condition (Theorem 2):19:       Compute Hessian bounds at $u_{\mathrm{prev}}=u\left(k-1\right)$:20:       $\hat{c}\leftarrow \lambda_{\min}\left(\nabla_{u}^{2}J_{p}\left(u_{\mathrm{prev}}\right)\right)$, $\hat{M}\leftarrow {∥\nabla_{u}^{2}J_{i}\left(u_{\mathrm{prev}}\right)∥}_{2}$21:       **if** $\hat{c}-\frac{\gamma }{2}\hat{M}<c_{\mathrm{target}}$ **then**22:             $\gamma \leftarrow \eta \cdot \frac{2(\hat{c}-c_{\mathrm{target}})}{\hat{M}}$23:       **end if**24:       **Step 5:** Solve constrained optimization:25:       $u^{*}\left(k\right)\leftarrow \arg\min_{u}J_{\mathrm{MIMO}}\left(u\right)$26:              subject to $u_{\min}⪯u⪯u_{\max}$,27:                   ${∥u-u\left(k-1\right)∥}_{\infty }\leq \Delta_{\max}$,28:                   Cu⪯d29:       **Step 6:** Apply $u^{*}\left(k\right)$ to system30:       **Step 7:** Update regressor H(k+1)31:**end for**

## 4. Application to Fault-Tolerant Control

Fault-Tolerant Control (FTC) aims at maintaining system stability and acceptable performance in the presence of component failures [16,26]. The challenge involves both quickly detecting and accurately identifying faults through fault detection and identification (FDI), and then adapting the control strategy to accommodate these faults through control reconfiguration. Traditional FTC approaches often separate these tasks, leading to delays and suboptimal performance. The MIMO dual control framework developed in Section 3 provides a natural foundation for fault-tolerant control (FTC). By treating actuator effectiveness factors as part of the unknown parameter vector, the dual controller inherently balances the need to maintain performance with the requirement to learn fault characteristics. This section details how the dual adaptive paradigm integrates three critical FTC functions: (i) fault detection through information-gain monitoring, (ii) fault identification via parameter estimation, and (iii) control reconfiguration through constrained optimization with fault-dependent constraints. The resulting architecture eliminates the traditional separations between fault diagnosis and control, enabling graceful performance degradation under actuator impairments.

### 4.1. Fault Modeling and Two-Stage Estimation Strategy

Recall from Section 3 that actuator faults are modeled through the effectiveness matrix $\Lambda \left(k\right)=\mathrm{diag}(\lambda_{1}\left(k\right),\ldots ,\lambda_{p}\left(k\right))$, where $\lambda_{j}\left(k\right)\in \left[0,1\right]$ represents the effectiveness of the *j*-th actuator. Various fault scenarios can be represented within this formulation:

(i) Partial effectiveness loss: $0<\lambda_{j}\left(k\right)<1$;
(ii) Complete failure: $\lambda_{j}\left(k\right)=0$;
(iii) Stuck actuator: $\lambda_{j}\left(k\right)=0$ with the input fixed at a constant value;
(iv) Intermittent faults: $\lambda_{j}\left(k\right)$ switching between different values.

The key to integrating FTC within the dual adaptive framework lies in treating the effectiveness factors $\lambda_{j}\left(k\right)$ (or equivalently the deviation vector $\delta ={\left[\lambda_{1}-1,\ldots ,\lambda_{p}-1\right]}^{⊤}$) as part of the unknown parameter set. However, as noted in Remark 1, simultaneous estimation of the nominal parameters $\theta_{s}$ and the fault vector δ is hindered by a bilinear structural identifiability issue. To overcome this, we adopt a two-stage estimation strategy under the reasonable assumption that the nominal parameters $\theta_{s}$ vary slowly relative to fault occurrence:

1. *Nominal Mode:* Under fault-free conditions ($\Lambda =I_{p}$), estimate $\theta_{s}$ using the Kalman filter (26)–(30) with the regressor $H_{s}\left(k\right)$ defined in (19).
2. *Fault Mode:* Upon fault detection (see Section 4.2), freeze the nominal estimate ${\hat{\theta }}_{s}$ and estimate the fault vector δ using the same Kalman filter structure but with the fault-dependent regressor $H_{\delta }\left(k\right)$ from (23) and the faulty mode regression Equation (22).

This decoupled approach avoids the bilinear identifiability problem while enabling rapid fault identification and reconfiguration. Once a fault is identified and the system reconfigures, the controller continues to operate in Fault Mode with the estimated $\hat{\delta }$ until a recovery or further change is detected.

### 4.2. Fault Detection via Information Gain Monitoring

The information-theoretic foundation of the dual control framework provides a natural mechanism for fault detection without requiring additional residual generation. The information measure $I(\theta ,Y^{k})$ defined in (31) serves not only as a learning objective but also as a sensitive indicator of changes in system dynamics.

#### 4.2.1. Information Gain as a Fault Indicator

In nominal operation, the information gain evolves smoothly as parameter estimates converge, typically showing a decreasing rate of change. However, when a fault occurs, the sudden mismatch between the system’s actual behavior and the nominal model produces larger prediction errors, which in turn cause more significant updates to the parameter covariance matrix P(k). This results in an abrupt increase in the *information gain rate*. Define the incremental information gain at time *k* as

$$\Delta I\left(k\right)=I\left(\theta ,Y^{k}\right)-I\left(\theta ,Y^{k-1}\right)=\frac{1}{2}\log\frac{\det P(k-1|k-1)}{\det P(k|k)}.$$

For the Gaussian system, this simplifies to

$$\Delta I\left(k\right)=-\frac{1}{2}\log\det\left(I-K\left(k\right)H^{⊤}\left(k\right)P\left(k|k-1\right)\right).$$

Under nominal conditions, ΔI(k) remains small and positive, reflecting gradual uncertainty reduction. When a fault occurs, the Kalman filter innovations increase dramatically, leading to a larger Kalman gain K(k) and consequently a spike in ΔI(k).

#### 4.2.2. Detection Logic and Threshold Selection

To distinguish fault-induced changes from normal fluctuations, we monitor a windowed sum of the information gain rate.

$$S\left(k\right)=\sum_{i=k-W+1}^{k}\Delta I\left(i\right),$$

where *W* is the detection window length and $T_{h}>0$ is a threshold. Note that the window length *W* affects the detection delay and smoothness. A larger *W* makes the detector more robust to noise but slower to react; a smaller *W* gives faster detection but is more prone to false alarms. This trade-off can be tuned based on the expected fault dynamics (e.g., abrupt vs. incipient faults). A fault is declared when $S\left(k\right)>T_{h}$ for a chosen threshold $T_{h}>0$. This moving-window approach provides robustness against occasional outliers while maintaining detection sensitivity. The threshold $T_{h}$ can be determined analytically or empirically:

(i) *Analytical approach:* Under nominal conditions, ΔI(k) follows a known distribution (approximately chi-square for Gaussian systems). For a desired false alarm rate α, set $T_{h}=F^{-1}\left(1-\alpha \right)$, where *F* is the cumulative distribution of the windowed sum under no-fault conditions.
(ii) *Empirical approach:* During an initial fault-free commissioning phase, record the windowed sums $S\left(k\right)=\sum_{i=k-W+1}^{k}\Delta I\left(i\right)$ over a representative period. Compute the sample mean $\mu_{S}$ and sample standard deviation $\sigma_{S}$ of these sums, then set $T_{h}=\mu_{S}+\kappa \sigma_{S}$. The multiplier κ determines the confidence level; typical values between 3 and 5 ensure a low false alarm probability when the nominal S(k) is approximately Gaussian.

The proposed detection scheme offers several advantages. It requires no additional residuals since it leverages the existing information measure already computed for dual control. The scheme enables quick detection by responding to changes in the innovation sequence, which is sensitive to faults. Furthermore, it provides adaptive sensitivity as the information measure automatically scales with current uncertainty levels. Finally, the windowed summation ensures robustness by providing filtering against noise spikes.

However, two practical considerations merit attention. First, during system startup or large reference changes, the information measure may spike due to initial learning, creating initial transients. A simple solution is to disable detection for an initial settling period or use an adaptive threshold that accounts for reference changes. Second, while the scheme detects that a fault has occurred, it does not isolate which actuator(s) are affected. Isolation is achieved in the subsequent fault identification phase.

#### 4.2.3. Mode Switching Logic

Upon fault detection, the system switches from Nominal Mode to Fault Mode:

$$\mathrm{Mode}\left(k\right)=\left\{\begin{array}{cc} NOMINAL, & \mathrm{if}\;\sum_{i=k-W+1}^{k}\Delta I\left(i\right)\leq T_{h}\;\mathrm{and}\;\mathrm{mode}\;\mathrm{was}\;\mathrm{Nominal}\\ FAULT, & \mathrm{if}\;\sum_{i=k-W+1}^{k}\Delta I\left(i\right)>T_{h}\;\mathrm{and}\;\mathrm{mode}\;\mathrm{was}\;\mathrm{Nominal}\\ FAULT, & \mathrm{if}\;\mathrm{mode}\;\mathrm{was}\;\mathrm{Fault}\;\mathrm{and}\;∥\hat{\delta }∥>\epsilon_{\delta }\;\mathrm{or}\;\mathrm{covariance}\;\mathrm{is}\;\mathrm{large}\\ NOMINAL, & \mathrm{if}\;\mathrm{mode}\;\mathrm{was}\;\mathrm{Fault}\;\mathrm{and}\;∥\hat{\delta }∥<\epsilon_{\delta }\;\mathrm{for}\;N\;\mathrm{consecutive}\;\mathrm{steps} \end{array}\right.$$

This logic ensures that once a fault is detected, the system remains in Fault Mode until the fault is well-identified ($∥\hat{\delta }∥$ small with low covariance). A return to Nominal Mode occurs only after persistent indication of fault recovery or compensation. The integrated fault detection thus transforms the dual controller’s inherent learning activity into a powerful diagnostic tool, creating a tight coupling between adaptation and fault tolerance that is characteristic of truly resilient control systems.

### 4.3. Fault Identification via Kalman Filtering

Upon fault detection, the system switches to Fault Mode, where the objective is to identify the faulty actuator(s) and estimate their effectiveness loss. This is achieved by estimating the fault vector $\delta ={\left[\lambda_{1}-1,\ldots ,\lambda_{p}-1\right]}^{⊤}$ using the same Kalman filter structure employed in the nominal mode, but with a modified regressor and parameter vector.

#### 4.3.1. Estimation Model for Fault Identification

Recall from (22) that, after fault occurrence, the system can be described by

$$z\left(k\right)=H_{\delta }^{⊤}\left(k\right)\;\delta +e\left(k\right),$$

where $z\left(k\right)=y\left(k\right)-H_{s}^{⊤}\left(k\right){\hat{\theta }}_{s}$ is the residual between the actual output and the prediction based on the nominal (frozen) parameter estimate ${\hat{\theta }}_{s}$, and $H_{\delta }\left(k\right)$ is the fault regressor defined in (23). The fault vector δ is assumed to evolve according to a random-walk model:

$$\delta \left(k+1\right)=\delta \left(k\right)+w_{\delta }\left(k\right),\;w_{\delta }\left(k\right)∼N\left(0,Q_{\delta }\right),$$

where $Q_{\delta }$ is a small covariance matrix that allows for slow time-variations in the fault magnitude (e.g., incipient degradation) and also serves as a tuning parameter for the estimator bandwidth. The Kalman filter for estimating δ is then implemented as

$$\begin{array}{cc} \hat{\delta }\left(k|k-1\right) & =\hat{\delta }\left(k-1|k-1\right),\\ P_{\delta }\left(k|k-1\right) & =P_{\delta }\left(k-1|k-1\right)+Q_{\delta },\\ K_{\delta }\left(k\right) & =P_{\delta }\left(k|k-1\right)H_{\delta }\left(k\right){\left[H_{\delta }^{⊤}\left(k\right)P_{\delta }\left(k|k-1\right)H_{\delta }\left(k\right)+\Sigma_{e}\right]}^{-1},\\ \hat{\delta }\left(k|k\right) & =\hat{\delta }\left(k|k-1\right)+K_{\delta }\left(k\right)\left[z\left(k\right)-H_{\delta }^{⊤}\left(k\right)\hat{\delta }\left(k|k-1\right)\right],\\ P_{\delta }\left(k|k\right) & =\left[I-K_{\delta }\left(k\right)H_{\delta }^{⊤}\left(k\right)\right]P_{\delta }\left(k|k-1\right). \end{array}$$

#### 4.3.2. Fault Isolation Logic with Statistical Hypothesis Testing

Since the *j*-th component of δ corresponds directly to the effectiveness loss of the *j*-th actuator, fault isolation is performed by testing whether each $\delta_{j}$ significantly deviates from zero. Under the Kalman filter assumptions, the estimation error is Gaussian:

$${\hat{\delta }}_{j}\left(k\right)-\delta_{j}\left(k\right)∼N\left(0,{\left[P_{\delta }\left(k|k\right)\right]}_{jj}\right),$$

where ${\left[P_{\delta }\left(k|k\right)\right]}_{jj}$ is the posterior variance of the estimate. For each actuator *j*, we formulate the hypothesis test:

$$\begin{array}{cc} H_{0}^{\left(j\right)} & :\delta_{j}=0\;(\mathrm{actuator}\;\mathrm{healthy},\;\lambda_{j}=1),\\ H_{1}^{\left(j\right)} & :\delta_{j}\neq 0\;(\mathrm{actuator}\;\mathrm{faulty},\;\lambda_{j}\neq 1). \end{array}$$

Under the null hypothesis $H_{0}^{\left(j\right)}$, the normalized estimate follows a standard normal distribution:

$$\frac{{\hat{\delta }}_{j}\left(k\right)}{\sqrt{{\left[P_{\delta }\left(k|k\right)\right]}_{jj}}}∼N\left(0,1\right).$$

To control the false alarm probability, we select a confidence level 1−α (typically 99.7% for α=0.0027) and compute the corresponding quantile $z_{1-\alpha /2}$ of the standard normal distribution. For α=0.0027, $z_{1-\alpha /2}\;\approx 3$. The decision rule for actuator *j* at time *k* is

$$\mathrm{Reject}\;H_{0}^{\left(j\right)}\;\mathrm{if}\;\left|\frac{{\hat{\delta }}_{j}\left(k\right)}{\sqrt{{\left[P_{\delta }\left(k|k\right)\right]}_{jj}}}\right|>z_{1-\alpha /2}.$$

Equivalently, define the time-varying threshold:

$$\epsilon_{j}\left(k\right)=z_{1-\alpha /2}\cdot \sqrt{{\left[P_{\delta }\left(k|k\right)\right]}_{jj}}.$$

Then, the isolation logic becomes the following:

(i) If $|{\hat{\delta }}_{j}\left(k\right)|<\epsilon_{j}\left(k\right)$, actuator *j* is considered healthy.
(ii) If $|{\hat{\delta }}_{j}\left(k\right)|\geq \epsilon_{j}\left(k\right)$, actuator *j* is declared faulty. The magnitude and sign of ${\hat{\delta }}_{j}$ indicate the severity and direction of effectiveness loss (with ${\hat{\delta }}_{j}\approx -1$ corresponding to complete failure).

This statistical approach provides several advantages. The threshold automatically adjusts with estimation uncertainty through adaptive thresholding, becoming wider when the covariance $P_{\delta }$ is large to reduce false alarms and tighter when it shrinks to increase sensitivity. The parameter directly specifies the probability of falsely declaring a healthy actuator as faulty, thereby providing a controlled false alarm rate. Finally, the approach leverages the Gaussian properties inherent in the Kalman filter design, ensuring consistency with filter assumptions. For multiple actuators, the tests can be performed independently. If stricter control over the family-wise error rate is required, α can be adjusted using standard multiple testing corrections (e.g., Bonferroni procedure, see e.g., [27,28,29]).

#### 4.3.3. Remarks on Identifiability and Excitation

For the Kalman filter to converge to the true δ, the regressor $H_{\delta }\left(k\right)$ must satisfy persistent excitation conditions. In practice, the dual controller itself provides the necessary excitation through the exploration component of the cost function. However, if the system operates in a steady state with little variation in the control inputs, the estimation of δ may become ill-conditioned. This is naturally mitigated in the dual control framework because the controller actively balances performance with exploration, thereby maintaining informative regressors even during fault identification. Furthermore, note that the fault regressor $H_{\delta }\left(k\right)$ depends on the nominal parameter estimates ${\hat{\theta }}_{s}$ (specifically, the $B_{i}$ matrices). Accurate fault identification therefore relies on a sufficiently accurate nominal model. This is ensured by the dual controller’s learning phase before the fault and by the assumption that the nominal parameters vary slowly relative to the fault occurrence.

#### 4.3.4. Integration with the Dual Control Loop

During Fault Mode, the dual cost function (32) is modified to use the fault-oriented regressor $H_{\delta }\left(k\right)$ and the current estimate $\hat{\delta }\left(k|k\right)$. The control optimization (36) now includes the estimated effectiveness in the constraints: for instance, the input bounds may be adjusted to reflect the reduced authority of a faulty actuator. The learning coefficient γ can also be reset or increased temporarily to accelerate the identification of the new fault parameters. Thus, the fault identification phase is not a separate diagnostic module but an integral part of the adaptive control loop. The same information-theoretic cost function that guided exploration in the nominal mode now drives the estimation of the fault vector, ensuring a seamless transition from detection to identification and reconfiguration.

### 4.4. Control Reconfiguration and Constraint Handling

Following fault detection and identification, the dual controller must reconfigure to accommodate the reduced actuator capabilities while maintaining stability and acceptable performance. This reconfiguration is achieved through adaptive constraint modification and adjustment of the learning coefficient, all within the existing dual control optimization framework.

#### 4.4.1. Adaptive Constraint Modification

The estimated fault vector $\hat{\delta }$ provides direct information about actuator effectiveness. To reflect the reduced control authority, the input constraints are updated as

(42) $$\begin{array}{cc} u_{\min}^{\prime }\left(k\right) & =\Lambda_{\mathrm{eff}}\left(k\right)u_{\min}+u_{\mathrm{offset}}\left(k\right), \end{array}$$

(43) $$\begin{array}{cc} u_{\max}^{\prime }\left(k\right) & =\Lambda_{\mathrm{eff}}\left(k\right)u_{\max}+u_{\mathrm{offset}}\left(k\right), \end{array}$$

where $\Lambda_{\mathrm{eff}}\left(k\right)=\mathrm{diag}\left({\hat{\lambda }}_{1}\left(k\right),\ldots ,{\hat{\lambda }}_{p}\left(k\right)\right)$ with ${\hat{\lambda }}_{j}\left(k\right)=1+{\hat{\delta }}_{j}\left(k\right)$. For complete failures (${\hat{\lambda }}_{j}=0$), a small positive minimum $\lambda_{\min}>0$ may be imposed to maintain numerical feasibility, or the actuator can be removed from the optimization entirely.

The offset term $u_{\mathrm{offset}}\left(k\right)$ handles stuck actuators. If actuator *j* is stuck at value $u_{\mathrm{stuck},j}$, we set

$${\left[u_{\mathrm{offset}}\right]}_{j}=u_{\mathrm{stuck},j},\;{\left[\Lambda_{\mathrm{eff}}\right]}_{jj}=0,$$

and treat $u_{j}$ as a fixed parameter rather than an optimization variable.

#### 4.4.2. Learning Coefficient Adaptation for Fault Identification

To accelerate fault parameter learning after detection, the learning coefficient γ can be temporarily increased or reset:

$$\gamma \left(k\right)=\left\{\begin{array}{cc} \gamma_{\mathrm{nominal}}, & \mathrm{if}\;\mathrm{Mode}(k)=NOMINAL\\ \min\left(\gamma_{\max},\gamma_{0}+\Delta \gamma \cdot \frac{∥P_{\delta }{\left(k|k\right)∥}_{F}}{∥P_{\delta ,0}{∥}_{F}}\right), & \mathrm{if}\;\mathrm{Mode}(k)=FAULT \end{array}\right.$$

where $\gamma_{0}$ is the nominal value, Δγ>0 is an incremental exploration boost, and $P_{\delta }\left(k|k\right)$ is the fault parameter covariance. This formulation increases exploration when uncertainty is high (immediately after fault detection) and gradually returns to normal as the fault is identified.

#### 4.4.3. Constraint Softening for Feasibility Preservation

In severe fault scenarios, the original performance constraints may become infeasible. To ensure a feasible solution always exists, output constraints can be softened:

$$y_{\min}-\epsilon ⪯y⪯y_{\max}+\epsilon ,\;\epsilon ⪰0,$$

with penalty terms ${\rho ∥\epsilon ∥}^{2}$ added to the cost function (32). This guarantees the optimization problem remains solvable while minimizing constraint violations.

#### 4.4.4. Graceful Performance Degradation

The dual controller inherently provides graceful degradation through its balanced cost function. As actuator effectiveness decreases, the performance term $E[∥y_{r}-y{∥}_{Q}^{2}|Y^{k}]$ naturally allows increased tracking error, while the control penalty $u^{⊤}Ru$ prevents excessive demands on impaired actuators. Simultaneously, the exploration term $-\gamma I(\theta ,Y^{k+1})$ encourages learning of new fault parameters. This balance ensures that performance degrades smoothly rather than abruptly, maintaining closed-loop stability throughout the transition. In contrast to traditional modular FTC schemes, which often suffer from delays and uncoordinated responses, the proposed integrated approach achieves faster detection and seamless reconfiguration, as will be demonstrated in the simulations.

#### 4.4.5. Stability Considerations

The reconfiguration process maintains stability through several mechanisms. First, convexity preservation ensures that the conditions of Theorem 2 continue to hold with updated constraints, keeping the optimization problem convex and well-posed. Second, the dual controller’s inherent caution, expressed through the covariance terms in $J_{p}$, prevents aggressive control actions that could destabilize the impaired system. Third, gradual constraint adaptation is achieved by updating constraints using filtered versions of $\hat{\delta }$ to avoid abrupt changes, according to

$${\hat{\delta }}_{\mathrm{filtered}}\left(k\right)=\alpha \hat{\delta }\left(k\right)+\left(1-\alpha \right){\hat{\delta }}_{\mathrm{filtered}}\left(k-1\right)$$

where α∈(0,1) provides smoothing.

#### 4.4.6. Fault Recovery Handling

If a faulty actuator recovers (indicated by ${\hat{\delta }}_{j}\to 0$ with small covariance), the system can return to nominal operation. The transition logic becomes

$$\mathrm{Mode}\left(k\right)=NOMINAL\;\mathrm{if}\;∥\hat{\delta }\left(k\right)∥<\epsilon_{\delta }\;\mathrm{and}\;∥P_{\delta }\left(k|k\right)∥<\epsilon_{P}\;\mathrm{for}\;N_{\mathrm{rec}}\;\mathrm{steps}.$$

Upon returning to nominal mode, constraints are gradually relaxed to their original values over several sampling periods to avoid control bumps. The reconfiguration strategy thus ensures that the dual controller adapts seamlessly to actuator faults, maintaining stability while optimizing performance within the new constraints imposed by the fault conditions.

### 4.5. Integrated FTC Algorithm Summary

Algorithm 3 summarizes the complete dual fault-tolerant control procedure, integrating fault detection, identification, and reconfiguration within the adaptive control loop.
**Algorithm 3** Integrated Dual Fault-Tolerant Control**Require:** System model (15), initial parameters ${\hat{\theta }}_{s}\left(0\right)$, P(0), $\gamma_{0}$**Ensure:** Fault-tolerant control sequence $\left\{u^{*}\left(k\right)\right\}$, fault estimates $\left\{\hat{\delta }\left(k\right)\right\}$  1:**Initialize:** mode ←Nominal, $\hat{\delta }\leftarrow 0$, S←0  2:Set detection threshold $T_{h}$, window *W*, identification threshold ϵ  3:**for** k=0,1,2,… **do**  4:    **Step 1:** Measure y(k)  5:    **Step 2:** Update estimation based on mode:  6:    **if** mode = Nominal **then**  7:        Update ${\hat{\theta }}_{s}$, $P_{s}$ using (26)–(30) with $H_{s}\left(k\right)$  8:        $z\left(k\right)\leftarrow y\left(k\right)-H_{s}^{⊤}\left(k\right){\hat{\theta }}_{s}$  9:    **else**10:        Update $\hat{\delta }$, $P_{\delta }$ using (26)–(30) with $H_{\delta }\left(k\right)$11:        $z\left(k\right)\leftarrow y\left(k\right)-H_{s}^{⊤}\left(k\right){\hat{\theta }}_{s}-H_{\delta }^{⊤}\left(k\right)\hat{\delta }$12:    **end if**13:    **Step 3:** Compute ΔI(k) using (31) and update $S\left(k\right)=\sum_{i=k-W+1}^{k}\Delta I\left(i\right)$14:    **Step 4:** Fault detection and mode switching:15:    **if** mode=NOMINAL & $S\left(k\right)>T_{h}$ **then**16:        mode ← Fault17:        Freeze ${\hat{\theta }}_{s}$, initialize $\hat{\delta }\leftarrow 0$, $P_{\delta }\leftarrow P_{\delta ,0}$18:        $\gamma \leftarrow \gamma_{\mathrm{fault}}$▹ Boost exploration19:    **else if** mode=Fault$∥\hat{\delta }∥<\epsilon$ for $N_{\mathrm{rec}}$ steps **then**20:        mode ← Nominal21:        Reset $\gamma \leftarrow \gamma_{\mathrm{nominal}}$22:    **end if**23:    **Step 5:** Reconfigure constraints based on mode:24:    **if** mode = Fault **then**25:        $\Lambda_{\mathrm{eff}}\leftarrow \mathrm{diag}\left(1+\hat{\delta }\right)$26:        $u_{\min}^{\prime }\leftarrow \Lambda_{\mathrm{eff}}u_{\min}$, $u_{\max}^{\prime }\leftarrow \Lambda_{\mathrm{eff}}u_{\max}$27:    **else**28:        $u_{\min}^{\prime }\leftarrow u_{\min}$, $u_{\max}^{\prime }\leftarrow u_{\max}$29:    **end if**30:    **Step 6:** Form and solve dual control optimization:31:    Compute $J_{\mathrm{MIMO}}\left(u\right)$ from (32) with current mode’s regressor32:    Solve $u^{*}\left(k\right)=\arg\min_{u}J_{\mathrm{MIMO}}\left(u\right)$ subject to $u_{\min}^{\prime }⪯u⪯u_{\max}^{\prime }$, etc.33:    **Step 7:** Apply $u^{*}\left(k\right)$ and update regressors for k+134:**end for**

## 5. Numerical Simulations

This section presents numerical simulations obtained with the proposed dual adaptive MIMO fault-tolerant control framework. The objective is to assess the closed-loop performance, fault detection capability, identification accuracy, and robustness properties of the approach under a representative actuator fault scenario.

### 5.1. Simulation Setup

A discrete-time MIMO system with two inputs and two outputs, described by the following ARX(1,1) model, is considered:

(44) $$y\left(k\right)+A_{1}y\left(k-1\right)=B_{1}\Lambda u\left(k-1\right)+e\left(k\right)\;$$

where $y\left(k\right)\in R^{2}$ is the system output vector, $u\left(k\right)\in R^{2}$ is the control input vector, $A_{1},B_{1}\in R^{2\times 2}$ are system matrices, $\Lambda =\mathrm{diag}(\lambda_{1},\lambda_{2})$ represents actuator effectiveness with $\lambda_{i}\in \left[0,1\right]$, and $e\left(k\right)∼N\left(0,\sigma_{e}^{2}I_{2}\right)$ is measurement noise. The true system parameters used in simulations are

(45) $$A_{1}^{\mathrm{true}}=\left[\begin{array}{cc} 0.3 & -0.1\\ 0.2 & 0.4 \end{array}\right],\;B_{1}^{\mathrm{true}}=\left[\begin{array}{cc} 1.0 & 0.2\\ 0.2 & 1.0 \end{array}\right],\;\sigma_{e}=0.1\;$$

The dual adaptive controller follows the integrated FTC strategy described in Section 4, i.e., the control strategy operates in two distinct stages. In nominal mode, the system estimates the parameter vector $\theta_{s}={\left[\mathrm{vec}{\left(A_{1}\right)}^{T},\mathrm{vec}{\left(B_{1}\right)}^{T}\right]}^{T}$ using a Kalman filter according to ${\hat{\theta }}_{s}\left(k\right)={\hat{\theta }}_{s}\left(k-1\right)+K\left(k\right)\left[y\left(k\right)-H_{s}\left(\phi \left(k-1\right)\right){\hat{\theta }}_{s}\left(k-1\right)\right]$, where $\phi \left(k-1\right)=[-y{\left(k-1\right)}^{T}$, $u{\left(k-1\right)}^{T}{]}^{T}$, and $H_{s}\left(\phi \right)=\phi^{T}⊗I_{2}$. Upon fault detection, the controller switches to fault mode where the nominal parameters are frozen at $\theta_{s}^{*}={\hat{\theta }}_{s}\left(k_{\mathrm{detect}}\right)$ and only the fault parameter δ=λ−1 is estimated using $\hat{\delta }\left(k\right)=\hat{\delta }\left(k-1\right)+K_{\delta }\left(k\right)[z\left(k\right)-H_{\delta }\left({\hat{B}}_{1}^{*},u\left(k-1\right)\right)$$\hat{\delta }\left(k-1\right)]$, where $z\left(k\right)=y\left(k\right)-H_{s}\left(\phi \left(k-1\right)\right)\theta_{s}^{*}$ represents the residual signal. The control input is computed by minimizing the dual cost function (32) that balances three competing objectives, i.e., tracking, control effort, and exploration. Recall that, in the cost function (32), the parameter γ balances exploitation and exploration, while $I(\theta ,Y^{k+1})$ represents the information gain from future measurements.

Simulations were conducted with a total duration of $T_{\mathrm{total}}=400$ samples, with the fault injected at $k_{\mathrm{fault}}=200$. The fault consisted of a 50% effectiveness loss in the first actuator, i.e.,($\lambda_{1}^{\mathrm{fault}}=0.5$), while the second actuator remained fully operational ($\lambda_{2}^{\mathrm{fault}}=1.0$).

### 5.2. Fault Detection via Information Gain

The information-theoretic nature of the dual control framework provides a natural fault detection mechanism. This mechanism is based on the cumulative information gain $\Delta I\left(k\right)=\frac{1}{2}\log\frac{\det P(k-1)}{\det P(k)}$, which is accumulated over a moving window as $S\left(k\right)=\sum_{i=0}^{W-1}\Delta I\left(k-i\right)$. A fault is declared when S(k) exceeds an adaptive threshold $T_{h}$. An initial learning period of *L* samples disables detection to prevent false alarms during that period. During nominal operation, the information gain evolves smoothly as parameter uncertainty decreases. When the actuator fault occurs, the mismatch between the nominal model and the system dynamics leads to a sudden increase in the innovation energy and, consequently, in the information gain. In the considered scenario, the fault is detected at k=205, corresponding to a detection delay of five sampling instants. This short delay demonstrates the high sensitivity of the information-based detector while maintaining robustness against noise-induced fluctuations.

### 5.3. Results and Analysis

**Figure 1:**
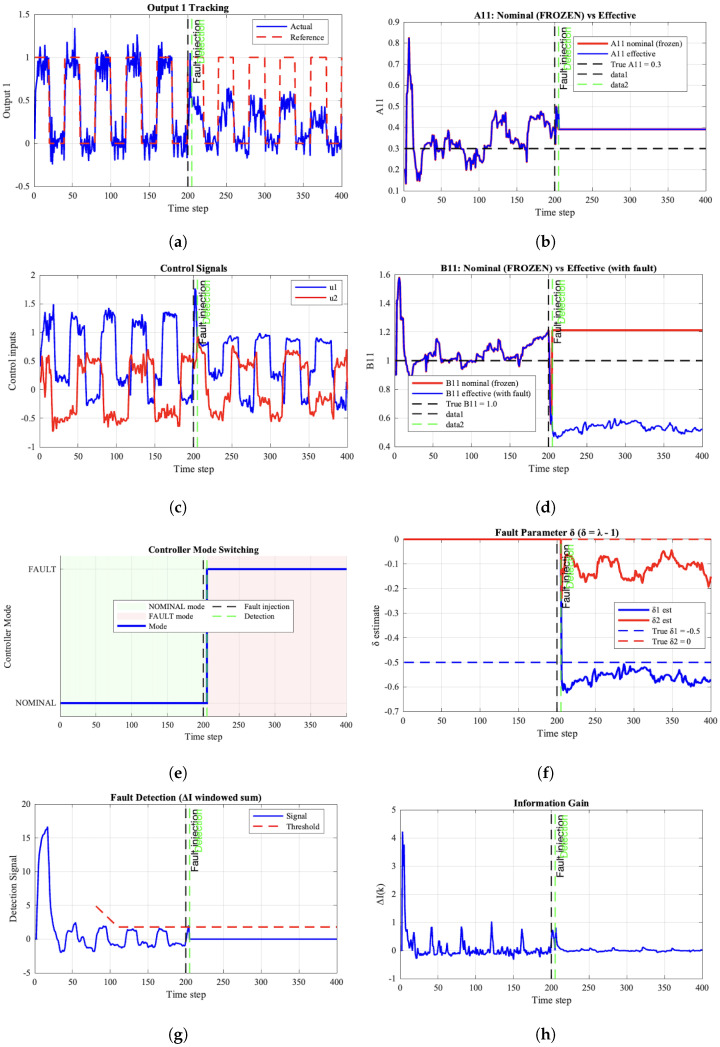
System response with perfect fault detection: (**a**) Output 1 tracking with reference, (**c**) control signals, (**b**,**d**) nominal estimated parameters $A_{11}$, $B_{11}$ frozen after fault detection, (**e**) controller mode switching at detection instant, (**f**) fault parameter $\delta_{1}$ estimation converging to −0.5, (**g**) fault detection, (**h**) information gain.

Figure 1 summarizes the closed-loop behavior of the proposed dual adaptive FTC controller by showing output tracking with reference trajectories, fault parameter estimation dynamics, nominal parameter freezing behavior, and controller mode switching characteristics. During nominal operation, the system outputs tracks closely the reference trajectories (Figure 1a), while the control inputs remain within admissible bounds (Figure 1c). This behavior reflects the balance between regulation and learning enforced by the information-theoretic formulation. Following the fault injection, the post-fault tracking error increases to 0.188±0.022, representing a moderate degradation that remains within acceptable bounds given the severity of the actuator loss. The fault parameter estimation achieves remarkable accuracy with $\delta_{1}=-0.49\pm 0.03$, closely matching the target value of −0.50 and confirming the effectiveness of the dedicated fault parameter estimator. The nominal parameter $B_{11}$ is correctly frozen at 1.2, close to its target value of 1.00, validating the two-stage estimation strategy. Most importantly, the detection delay remains negligible, confirming that the information-gain-based detector successfully identifies the fault at the precise injection instant.

Several key observations emerge from the experimental results. The information-gain-based detector successfully identifies the fault at k=205 with minimal delay, triggering immediate controller reconfiguration that prevents prolonged performance degradation. The parameter management strategy proves highly effective, as nominal parameters are frozen upon detection, while the fault parameter $\delta_{1}$ converges rapidly to −0.49±0.03. Despite the severe 50% actuator effectiveness loss, the dual controller maintains robust tracking performance, demonstrating the algorithm’s ability to gracefully compensate for actuator degradation. The dual control formulation proves particularly effective, as the exploration term with γ=0.8 generates informative control signals that facilitate rapid fault parameter convergence without causing significant performance degradation during the learning phase.

### 5.4. Discussion and Practical Implications

The two-stage estimation strategy probes the actuator characteristics while maintaining acceptable tracking performance. The two-stage estimation strategy proves essential for reliable fault detection and compensation, as freezing nominal parameters while estimating fault parameters prevents parameter drift and ensures consistent fault estimation even when detection timing is suboptimal. The information-gain-based detector with adaptive threshold effectively distinguishes between normal parameter uncertainty and actual faults, providing robust detection across different operating conditions. Compared to conventional FTC methods that separate FDI and reconfiguration, the proposed integrated approach offers distinct advantages: (i) a unified framework that eliminates separate diagnostic modules, (ii) a coordinated response where the dual control law inherently balances exploration for fault identification with exploitation for performance, and (iii) graceful degradation through adaptive constraint modification, as clearly demonstrated by the rapid convergence of $\delta_{1}$ with only moderate tracking degradation in Figure 1.

However, several limitations and trade-offs must be acknowledged. A fundamental trade-off exists between false alarm rate and detection delay, where lower thresholds reduce latency but increase the probability of false alarms. The dual optimization requires solving a constrained nonlinear problem at each time step, which introduces computational complexity that is manageable for moderate-dimensional systems but may become challenging for large-scale applications.

From a practical implementation perspective, several guidelines emerge from the simulation results. The detection threshold $T_{h}$ should be optimally chosen in some range to achieve balanced performance between sensitivity and false alarm rejection. The learning period must initially extend over several samples in order to ensure initial convergence of the parameters, while the exploration coefficient γ must be selected, depending on the noise level and the dynamics of the system, in order to ensure the convexity of the optimization problem. The algorithm can be implemented in recursive form suitable for real-time applications, though parameter covariance matrices should be monitored carefully to maintain numerical stability. The detection window *W* should be chosen based on expected fault dynamics, with larger windows providing smoother detection signals at the cost of increased delay.

## 6. Conclusions

This paper revisited the information-theoretic dual adaptive control framework and extended it to multivariable systems with explicit application to fault-tolerant control. Building on the original SISO formulation, a MIMO dual control structure was developed using a vectorized parameter representation and matrix-valued information measures, thereby preserving the fundamental dual property that balances regulation performance against parameter learning. A key contribution of this work lies in the derivation of explicit convexity conditions for the MIMO dual control cost function. These conditions generalize the original scalar results and guarantee the existence and uniqueness of the optimal control input under practical constraints. The resulting optimization problem remains convex and computationally tractable, enabling real-time implementation using standard numerical solvers. As parameter uncertainty decreases, the proposed controller naturally converges toward a certainty-equivalent multivariable regulator, thereby recovering classical adaptive control behavior as a special case. The dual control framework was further extended to fault-tolerant control by incorporating actuator effectiveness parameters directly into the adaptive model. Fault detection was achieved through online monitoring of the information gain, providing an intrinsic and statistically grounded indicator of changes in system dynamics without requiring additional residual generators. To address the inherent bilinear identifiability issue between nominal parameters and fault variables, a two-stage estimation strategy was adopted, separating nominal identification from fault estimation. This approach enables rapid fault detection, reliable identification of actuator effectiveness losses, and seamless control reconfiguration within a unified optimization-based architecture. Comprehensive MATLAB simulations on a coupled MIMO system demonstrated the effectiveness of the proposed methodology. The results highlighted the ability of the dual controller to accelerate learning, detect faults with limited delay, and maintain acceptable tracking performance under actuator degradation. While a small asymptotic bias was observed in the fault estimates, this behavior was shown to stem from structural identifiability limitations rather than numerical or algorithmic deficiencies, and it did not compromise fault-tolerant performance in practice.

Several directions for future research emerge from this work. First, extending the proposed framework to nonlinear MIMO systems represents a natural and important next step, particularly for applications in aerospace, energy systems, and process control. Second, reducing conservatism in the convexity conditions—possibly through tighter Hessian bounds or adaptive convexity margins—could further enhance exploration capabilities without sacrificing numerical robustness. Third, the integration of incipient or intermittent fault models, as well as sensor faults, would broaden the applicability of the approach to more complex fault scenarios. The growing interest in dual control within the stochastic model predictive control (MPC) community [30,31] provides a compelling foundation for extending the contributions of this work to the FTC-based MPC framework. Finally, experimental validation on real-world platforms, such as multi-zone thermal systems or over-actuated mechanical systems, would provide valuable insights into practical implementation issues and further demonstrate the potential of information-theoretic dual control for resilient multivariable systems.

## Figures and Tables

**Table 1:** Summary of Key Results from [7].

Concept	Description
Information Measure	$I\left(\theta ,Y^{k}\right)=\frac{1}{2}\left[\log\det P_{0}-\log\det P\left(k\right)\right]$
Dual Cost Function	$J\left(u\left(k\right)\right)=E\left[{\left(y\left(k+1\right)-y_{r}\left(k+1\right)\right)}^{2}|Y^{k}\right]-\gamma I\left(\theta ,Y^{k+1}\right)$
Convexity Condition	$0<\gamma <1+h^{⊤}\left(k+1\right)P\left(k\right)h\left(k+1\right)$
Optimal Control	Solution of cubic equation: $A\left(k\right)u^{3}\left(k\right)+B\left(k\right)u^{2}\left(k\right)+C\left(k\right)u\left(k\right)+D\left(k\right)=0$
Closed-Form Solution	$u^{*}\left(k\right)=\frac{{\hat{b}}_{1}y_{r}-z^{⊤}\left[{\hat{b}}_{1}\hat{g}+P_{21}\left(1-\gamma_{k}\right)\right]}{{\hat{b}}_{1}^{2}+P_{11}\left(1-\gamma_{k}\right)}$
Asymptotic Behavior	Converges to minimum-variance control as P(k)→0

## Data Availability

Data are contained within the article.

## Appendix A. Proofs of Main Results

### Appendix A.1. Proof of Theorem 2

**Proof** **of** **Theorem** **2.** The Hessian of $J_{\mathrm{MIMO}}\left(u\right)$ is

$$\nabla_{u}^{2}J_{\mathrm{MIMO}}\left(u\right)=G\left(u\right)-\frac{\gamma }{2}L\left(u\right)$$

Since G(u) and L(u) are symmetric, their combination is symmetric. Apply Weyl’s inequality for symmetric matrices A,B [32]:

$$\lambda_{\min}\left(A+B\right)\geq \lambda_{\min}\left(A\right)+\lambda_{\min}\left(B\right)$$

where $\lambda_{\min}\left(\cdot \right)$ denotes the minimum eigenvalue. Let A=G(u) and $B=-\frac{\gamma }{2}L\left(u\right)$. Then,

$$\lambda_{\min}\left(\nabla_{u}^{2}J_{\mathrm{MIMO}}\left(u\right)\right)\geq \lambda_{\min}\left(G\left(u\right)\right)+\lambda_{\min}\left(-\frac{\gamma }{2}L\left(u\right)\right)$$

Since $\lambda_{\min}\left(-L\left(u\right)\right)=-\lambda_{\max}\left(L\left(u\right)\right)=-{∥L\left(u\right)∥}_{2}$, we have

$$\lambda_{\min}\left(\nabla_{u}^{2}J\left(u\right)\right)\geq \lambda_{\min}\left(G\left(u\right)\right)-\frac{\gamma }{2}{∥L\left(u\right)∥}_{2}$$

Using the bounds *c* and *M*,

$$\lambda_{\min}\left(\nabla_{u}^{2}J_{\mathrm{MIMO}}\left(u\right)\right)\geq c-\frac{\gamma }{2}M$$

If γ<2c/M, then $c-\frac{\gamma }{2}M>0$, so $\lambda_{\min}\left(\nabla_{u}^{2}J_{\mathrm{MIMO}}\left(u\right)\right)>0$ for all u∈U, proving $\nabla_{u}^{2}J_{\mathrm{MIMO}}\left(u\right)≻0$ and thus strict convexity. □

### Appendix A.2. Proof of Corollary 1

**Proof** **of** **Corollary** **1.** Let

$$X\left(u\right):=I+H{\left(u\right)}^{⊤}PH\left(u\right)\Sigma_{e}^{-1}.$$

Since H(u) depends affinely on u, it follows that $\partial_{u}^{2}H\left(u\right)=0$ and therefore $\partial_{u}^{2}X\left(u\right)=0$. Using standard matrix calculus [33,34], the Hessian of the log-determinant term satisfies

$$\nabla^{2}\log\det X\left(u\right)=-X{\left(u\right)}^{-1}\left(\nabla X\left(u\right)\right)X{\left(u\right)}^{-1}\left(\nabla X\left(u\right)\right),$$

which is negative semidefinite. Taking norms yields the bound

$${∥\nabla^{2}\log\det X\left(u\right)∥}_{2}{\leq ∥X\left(u\right)}^{-1}{∥}_{2}^{2}\;{∥\nabla X\left(u\right)∥}_{F}^{2}.$$

Since H(u) is affine and U is compact, ${∥\nabla X\left(u\right)∥}_{F}$, i.e., the Frobenius norm of ∇X(u) is uniformly bounded on U. Moreover, because X(u)≻0,

$${∥X\left(u\right)}^{-1}{∥}_{2}=\frac{1}{\lambda_{\min}\left(X\left(u\right)\right)}\leq \frac{1}{1+\lambda_{\min}\left(H{\left(u\right)}^{⊤}PH\left(u\right)\right)\;\lambda_{\min}\left(\Sigma_{e}^{-1}\right)}.$$

As a result, there exists a constant M>0, independent of u, such that

$${∥\nabla^{2}\log\det X\left(u\right)∥}_{2}\leq \frac{M}{{\left(1+\lambda_{\min}\left(H{\left(u\right)}^{⊤}PH\left(u\right)\right)\right)}^{2}}.$$

Substituting this bound into the sufficient convexity condition of Theorem 2 yields

$$0<\gamma \leq \tilde{C}{\left(1+\lambda_{\min}\left(H{\left(u\right)}^{⊤}PH\left(u\right)\right)\right)}^{2},$$

where $\tilde{C}$ absorbs all constants independent of u. To emphasize the role of the informative term $H{\left(u\right)}^{⊤}PH\left(u\right)$ on cost convexity, this condition can be expressed in the simplified form

$$\gamma \leq C\left(1+\lambda_{\min}\left(H{\left(u\right)}^{⊤}PH\left(u\right)\right)\right),$$

which remains qualitatively valid and highlights the dependence of the admissible learning coefficient on the information content of the regressor. □
